# Inequities in Household Out-Of-Pocket Spending Among Urban Slum Dwellers in Southeast Nigeria

**DOI:** 10.3389/ijph.2025.1607969

**Published:** 2025-03-27

**Authors:** Okechukwu Ozor, Chukwudi Nwokolo, Noemia Teixeira de Siqueira Filha, Aloysius Odii, Joseph Paul Hicks, Shushan Li, Uchenna Ezenwaka, Bryony Dawkins, Obinna Onwujekwe

**Affiliations:** ^1^ Health Policy Research Group, University of Nigeria, Enugu, Nigeria; ^2^ Department of Economics, University of Nigeria, Nsukka, Nigeria; ^3^ Department of Health Sciences, University of York, York, United Kingdom; ^4^ Department of Sociology and Anthropology, University of Nigeria, Nsukka, Nigeria; ^5^ Nuffield Centre for International Health and Development, Leeds Institute of Health Sciences, School of Medicine, University of Leeds, Leeds, United Kingdom; ^6^ Academic Unit of Health Economics, Leeds Institute of Health Sciences, University of Leeds, Leeds, United Kingdom

**Keywords:** ability to pay, inequity, Kakwani index, out-of-pocket expenditure, urban slum

## Abstract

**Objectives:**

This study examines the economic burden and inequities in out-of-pocket expenditures (OOPEs) to access healthcare in urban slums in Nigeria.

**Methods:**

The cross-sectional study was undertaken in eight urban slums in Enugu and Anambra, Nigeria. Participants (n = 1,025) responded to questions on health expenditures and access to healthcare. Gamma regression was used to estimate the mean differences in OOPE. Financing incidence analysis was used to estimate inequities in OOPE.

**Results:**

Enugu residents and individuals with formal occupations incurred lower costs than the residents in Anambra and those employed in informal occupations. Households in the middle wealth quintile incurred higher costs than those in the poorest quintile. Gini, concentration, and Kakwani indices indicated a progressive financing system, with the richest contributing proportionately more than their share of ability to pay (ATP). Poorest households used informal healthcare more.

**Conclusion:**

Although payment for healthcare in urban slums is progressive, the poorest households may be at risk of poor health outcomes due to reliance on informal healthcare providers. Our findings highlight the role ATP may play in healthcare denial among the urban poor.

## Introduction

High out-of-pocket expenditure (OOPE) has been described as an inequitable health financing mechanism across different countries, especially in low- and middle-income countries (LMICs) in sub-Saharan Africa [1–4]. This financing mechanism involves health consumers directly paying money that may or may not be reimbursed later while accessing healthcare services. While wealthier households may feel less impact after making these health expenditures, poorer households may have to forgo other important needs after such payments for the same services, thus incurring catastrophic health expenditures and further impoverishment. This points to the problem of inequity in accessing healthcare services.

To prevent this problem of inequity in healthcare access, one important goal of health systems is to promote fairness of financial contributions. This means that each household should contribute to the country’s health system according to their ability to pay (ATP), exempting impoverished households from making any payments [5]. This approach can help shield households from financial risks due to high health expenditures [6, 7].

Approximately 5 million Nigerians, representing only 3% of the population, have health insurance coverage through the country’s National Health Insurance Scheme (NHIS). A significant portion of these individuals are federal government employees rather than workers at the state or regional level [8, 9]. This leaves about 97% of the population uninsured, relying on out-of-pocket payments to access health services. The high level of OOPE has worsened the Nigerian health system and hindered progress towards achieving Universal Health Coverage [10].

### The Research Problem

Inequities in accessing healthcare services particularly affect the urban poor, whose unstable and informal work arrangements hinder their ability to access quality care and maintain good health [11, 12]. The failure to provide sufficient public primary care, alongside the presence of numerous private healthcare providers and considerable variations in service quality, poses major obstacles to effective health-seeking behavior [13]. The lack of reliable, affordable public services and the reliance on the private sector also contribute to exorbitant healthcare costs, resulting in catastrophic healthcare expenditures for the most vulnerable populations living in urban slums [14].

The urban population in Nigeria is expanding rapidly. The World Bank’s estimates indicate that in 2023, 54.28% of Nigeria’s population resided in urban areas [15]. According to the most recent Multidimensional Poverty Index Survey conducted in 2022, 42% of the urban population was multidimensionally poor [16]. Furthermore, the World Bank estimated that 49% of the total urban population lived in slums in 2020 [15].

Ensuring an effective allocation of resources and risk management systems to mitigate the burden of OOPE for urban slum dwellers requires that policymakers understand the intricacies of this economic burden. This is pertinent, considering that poor households readily suffer from catastrophic expenditures when prioritizing healthcare over basic household needs [17] Therefore, exploring the inequity in OOPE in urban slums and its predictor factors can provide more insights into the economic burden of healthcare access for urban slum dwellers and thus guide the design of effective interventions [3].

### Current Evidence and Gaps in the Literature

Globally, evidence shows that almost one billion individuals suffer catastrophic health expenditures annually, with 70 million individuals pushed to extreme poverty [18]. The highest concentration of catastrophic health expenditures has been in the world’s poorest regions – Asia and Africa, at 16.6% and 10.0%, respectively [18]. Households in poor countries that rely on OOPE as the main source of health financing are at higher risk of exacerbating poverty [1].

Nigeria follows the same pattern as other LMICs, where most households rely on OOPE for healthcare utilization [7]. OOPE is a key factor preventing Nigerians from accessing quality and formal healthcare services [19], and it has been shown to affect the availability of quality health services in the country [20]. This financing arrangement is unfair and exposes health consumers to financial risks. It also increases the non-affordability of health services, ultimately impacting health outcomes and coverage negatively [21].

The high level of OOPE has made access to healthcare difficult for the most vulnerable populations living in urban slums. A recent scoping review [14] on the economics of healthcare access for urban slum dwellers indicated high OOPE to access healthcare for acute and chronic conditions. Applying purchasing power parity, the costs to treat acute conditions varied from $157 to $408 for the poorest and wealthier quintiles, respectively. Costs for patients with chronic conditions varied from $720 to $1,470 for the poorest and wealthier quintiles, respectively [14].

There is a paucity of empirical evidence on how OOPE is distributed across socioeconomic groups relative to their ATP, especially amongst urban slum dwellers. It is noted that few studies in other parts of the world have explored the impact of OOPE on health across urban slum dwellers [14, 22, 23]. In Nigeria, Onwujekwe et al. [4] assessed the inequity in OOPE across urban and rural dwellers. They found that the poorest socio-economic group experienced the highest burden of OOPE, suggesting inequity in access to healthcare services among urban and rural dwellers. However, there is no evidence of inequity in healthcare expenditures for urban slums in Nigeria. Hence, this study intends to fill this gap by providing empirical evidence of this effect.

### Study Objectives

This paper aims to assess the level of health inequity in OOPE for different socio-economic groups among households in urban slums in Enugu and Anambra states in Nigeria.

Specifically, this paper aims to:

• Characterize the demographic and socio-economic characteristics of the study population.
• Characterize the health-seeking behavior of households in urban slums in Enugu and Anambra states.
• Estimate the association between OOPE in health, socio-economic status, and health-seeking behavior of households living in urban slums in Enugu and Anambra States.
• Explore inequity in healthcare expenditure among households living in urban slums in Enugu and Anambra States.

The findings will be useful for guiding policymakers to reduce the burden of OOPE in urban slums, especially amongst the poorest households, and to prevent them from suffering further impoverishment and lack of access to quality healthcare.

## Methods

### Study Design and Setting

We employed a cross-sectional survey design in this study as an efficient method to collect large amounts of data at once. In the current paper, we use data from the survey to inform OOPE on health among the different socio-economic groups in the urban slums in southeast Nigeria.

Nigeria is a West African country between the Sahel to the north and the Gulf of Guinea to the south in the Atlantic Ocean. The country has a staggering population of more than 229 million people in 2024, at an annual growth increase of 2.39%, making it the most populous country in Africa [24]. According to the United Nations [25], about 80 million Nigerians, representing 79% of the population, are living in slums. Our study was conducted in eight urban slums purposively sampled across two cities, Enugu and Onitsha, located in Enugu and Anambra states in southeast Nigeria in October 2022 (Enugu slums = Afia-Nine, Ngenevu, Ugbo-Oghe, and Ikilike; Anambra slums = Okpoko 4, Okpoko 5, Prison Marine, and Ibollo). Enugu and Anambra states were purposefully selected for the study because they are close to one another and the research team. Also, these states include several sizable, long-standing urban slums. According to Macrotrends [26, 27], Enugu City and Onitsha have an estimated population of 876,000 and 1,695,000, with an annual growth increase of 3.42% and 4.44%, respectively. Both states are predominantly inhabited by the Igbo tribe. Enugu is the capital city of the state of Enugu. The state is bordered to the south by Abia state, to the north by Benue and Kogi states, to the east by Ebonyi, and to the west by Anambra state. There are 17 Local Government Areas (LGAs) in Enugu state, of which five are largely urban. Onitsha is one of the commercial cities in Anambra state. The state is bordered to the south by Imo and Rivers state, north by Kogi state, west by Delta state, and the east by Enugu state. The capital city of Anambra is Awka. Anambra state comprises 21 LGAs, six of which are urban. In both Enugu and Onitsha cities, large areas of slums are scattered around mixed-income and impoverished neighborhoods. There is a lack of accessibility to fundamental urban public infrastructure, such as health facilities, in both cities [28].

### Study Population

The overall target population is the female primary caregivers of households containing at least one woman aged 15–49 and/or at least one child aged ≤5, within Nigeria slums. Participants were eligible if they were community members living in an eligible household within the study sites. Within eligible households, we primarily sought the main female caregiver as the eligible respondent, but if such an individual was not available then we allowed the male head of the household to be the eligible respondent if they had sufficient knowledge of household health-seeking behavior and expenditure.

### Sample Size Calculation and Sampling Method

The minimum sample size of 1,025 households was determined using the Demographic and Household Survey Sampling and Household Listing Manual sample size calculation formula [29].

$$n={Deft}^{2}\times \frac{\frac{1}{p}-1}{\alpha^{2}}$$

Since the survey was descriptive, with most of the results of the questionnaire responses in either binary or ordinal/multinomial outcomes, which in turn were treated as a series of binary outcomes in practice, our sample size calculation was based on the ability to estimate 95% confidence intervals for proportions relating to our binary outcomes. For the most conservative/generalizable proportion of 0.5, assuming a design effect (deft) of 1.6 (which was typical of indicators from the Nigerian Demographic and Health Survey 2018), and targeting a 95% confidence interval width of ±0.098 (based on a relative standard error of 0.05), a sample size of 1,025 households were estimated to be needed in our study. These 1,025 households were further shared equally among the eight slums since they are similar in size (resulting in approximately 128 households per slum).

The purposive sampling method was employed in this study based on convenience. Initially, we purposively selected three LGAs in each state based on population, density size, and diverse urban slum settings to ensure a representative sample. In addition, we purposefully selected a total of eight slums (four per state) across the local government areas, based on three criteria: 1) the relative size of the slum to ensure similarly sized slums in both states, 2) the slum being judged as suitably accessible and safe for our research team to work in, given local contextual considerations, and 3) the slum having at least one functional primary health center (PHC). See Table 1.

**TABLE 1:** Sampling details (Enugu and Anambra, Nigeria. 2022).

State (City)	No. of LGAs	Selected LGAs	No. slums	Selected slums
Enugu	17	Enugu North	12	Afia-Nine and Ngenevu
(Enugu)		Enugu East	6	Ugbo-Oghe
		Enugu South	3	Ikiriki
Anambra	21	Ogbaru	8	Okpoko 4 and Okpoko 5
(Onitsha)		Onitsha North	3	Prison marine
		Ekwusigo	1	Ibollo

LGA, local government area.

The data collection team began at the PHC in each slum, found the closest household, and attempted to speak with an eligible family member. They continued in this manner with the next closest household until the cluster sample size was reached. A home was skipped by the team if there were no willing or eligible participants.

### Study Method

An interviewer-administered questionnaire developed by the study team was used in data collection at the household level. The questionnaire was pre-tested in Coal Camp slum community in Enugu with six households. Data was collected in pairs, with one of the pairs using the paper questionnaire version and the other a tablet containing a soft copy of the questionnaire. The questionnaire elicited information on socio-economic characteristics, healthcare access, and OOPEs during their most recent healthcare visit within the 3 months before the interview. OOPE (direct medical costs: drugs, tests, consultation fees) was collected in the local currency, Nigerian Naira, and converted to US dollars by using the exchange rate for September 2022 (1 NGN = 430.99 USD, OANDA.com).

Following the completion of data collection daily, each pair cross-checked all answers for consistency between the paper and tablet versions of the questionnaire. At the same time, the field supervisor also performed an additional cross-check. After that, the electronic data was uploaded to the database, while the paper version was retained and kept confidential. The data manager next reviewed the information again to search for any anomalies while correcting it with the retained paper copies.

### Variable Measurements

#### Outcome or Dependent Variable

This is the variable that changes when the independent variable is manipulated. In the current study, the dependent variable is OOPE, measured as the amount of money spent by the household while seeking care that was not reimbursed.

#### Explanatory or Independent Variables

This is the variable that explains the change observed in the dependent variable. In this study, our independent variables can be broadly categorized into two:

o The socio-economic variables: these variables were measured as the status of the respondent in the household, the gender, the educational level, occupation or major source of income, the current marital status, and wealth quintiles where the household falls.
o Healthcare utilization variables: these variables were measured as the facility type where the household sought care, the health conditions treated, the nature of care received (whether outpatient or inpatient), and the main challenges the household encountered with accessing care at the health facilities.

### Statistical Analysis

The data was analyzed using Stata version 17. Descriptive statistics were used to describe the socio-economic characteristics of the study respondents by calculating frequencies and interquartile ranges for each state and overall. We also applied this approach to characterize healthcare access and utilization.

Gamma regression analysis was also undertaken to explore the association between OOPE and socio-economic characteristics (State, age, occupation, education, marital status, wealth quintile), and healthcare utilization characteristics (type of health service sought).

We adopted principles of financing incidence analysis to analyze inequities in OOPE by computing the Gini, concentration, and Kakwani indices. We first categorized households into quantiles of ATP. The household ATP was calculated by the household total monthly expenditures on food and non-food items such as education, clothing, rent, recreation/entertainment, cooking fuel, healthcare, and durable household goods (such as electrical equipment and furniture). For each quantile, an estimate of OOPE as a fraction of ATP total household expenditure per adult equivalent was computed. The Gini index was calculated to estimate the distribution of ATP across wealth quintiles, which was defined based on the survey sample. It was calculated by the cumulative percentage of ATP against the cumulative percentage of the population. The Gini index ranges from 0 to +1; the closer the index is to 0, the more equal the distribution of ATP. The concentration index computed the distribution of OOPE according to the wealth quintiles. The index varies from −1 and +1, with (−) denoting the concentration of expenditures amongst the poorest (regressivity) and (+) the concentration amongst the richest (progressivity). The Kakwani index was obtained as the difference between the concentration index of OOPE and the Gini index). The Kakwani index is bounded between −2.00 and 1.00, with a positive value indicating progressivity, and a negative value indicating regressivity of the health financing system [30, 31].

To investigate whether the finance incidence analysis indicates better/worse healthcare access, we tabulated wealth quintiles against the type of health access (e.g., formal only, informal only, and both formal and informal) presenting it in bar charts for each state and overall.

### Ethics Declaration

Ethical approval was obtained from the Health Research Ethics Committee of the University of Nigeria Teaching Hospital (NHREC/05/01/2008B-FWA00002458-IRB00002323) and the School of Medicine Research Ethics Committee of the University of Leeds, UK (reference: MREC 21- 009). The relevant guidelines and ethical principles ensuring the integrity and protection of participants were strictly adhered to by informing the participants verbally together with a written consent form containing information about the study, the risks and benefits of participating as well as the right to free withdrawal from the study at any time without any repercussion. This was explained and discussed with participants until they noted that they had understood and gave their consent (both verbally and written) before they were interviewed.

## Results

### Demographics and Socio-Economic Characteristics of the Study Participants

A total of 1,025 households were surveyed for the study, 509 in Anambra and 516 in Enugu (Table 2). In Enugu, most respondents (N = 375, 73%) were female heads of household, whereas in Anambra, other representatives of the household were the most frequent respondents (N = 393, 77%). The median age was similar in both states, with an overall median of 31 years (IQR: 27–37). Most of the respondents in both states (N = 741, 74%) indicated secondary education as their highest level of education. Overall, 81% (N = 827) of the respondents worked in informal jobs, while 12% (N = 125) were unemployed. Regarding marital status, 93% (N = 955) of the respondents in both states were currently living with their spouse/partner. Anambra state had more households in the poorest/poor wealth quintile than Enugu (47% vs. 33%).

**TABLE 2:** Socio-economic characteristics of participants (Enugu and Anambra, Nigeria. 2022).

Characteristics	Anambra N = 509	Enugu N = 516	Total N = 1,025
Status in Household, N (%)			
Female head of household	116 (22.79)	375 (72.67)	491 (47.90)
Male head of household	0 (0.00)	36 (6.98)	36 (3.51)
Other representatives of household ^a^	393 (77.21)	105 (20.35)	498 (48.59)
Gender, N (%)			
Female	508 (99.80)	475 (92.05)	983 (95.90)
Male	1 (0.20)	41 (7.95)	42 (4.10)
Median Age (IQR)	30 (27–36)	32 (27–38)	31 (27–37)
Education Level ^b^, N (%)			
Primary Education and less	70 (13.94)	47 (9.40)	117 (11.68)
Secondary Education	379 (75.50)	362 (72.40)	741 (73.95)
Tertiary Education	53 (10.56)	91 (18.20)	144 (14.37)
Occupation/Major Source of Income ^c^, N (%)			
Informal	413 (81.14)	414 (80.23)	827 (80.68)
Unemployed	62 (12.18)	63 (12.21)	125 (12.20)
Formal	34 (6.68)	39 (7.56)	73 (7.12)
Current Marital Status ^d^, N (%)			
Not currently married	14 (2.75)	56 (10.85)	70 (6.83)
Currently Married	495 (97.25)	460 (89.15)	955 (93.17)
Median number of people in the household (IQR)	5 (4–7)	5 (4–6)	5 (4–6)
Wealth quintile, N (%)			
Poorest	110 (21.61)	95 (18.41)	205 (20.00)
Less poor	127 (24.95)	78 (15.12)	205 (20.00)
Middle	138 (27.11)	67 (12.98)	205 (20.00)
Rich	106 (20.83)	99 (19.19)	205 (20.00)
Richest	28 (5.50)	177 (34.30)	205 (20.00)

^a^ Other household representative = any other person representing the household other than the female and male head of household, e.g., wife, grandmother, etc.

^b^ Education Level: Primary education or less = some primary, primary; secondary education = junior secondary, senior secondary; tertiary education = university, and teachers training college.

^c^ Occupation/Major Source of Income: Informal = subsistent farming, petty trading, artisanal worker, owners of big businesses, and self-employed; unemployed = unemployed, students, and housewives; formal = pensioner, government worker, and employed in the formal private sector with a salary.

^d^ Current Marital Status: Not currently married = never married, widowed, and divorced/separated; Currently married.

= living with spouse/partner.

### The Health-Seeking Behavior of Households Living in Urban Slums in Enugu and Anambra States

In total, 892 (87%) respondents reported utilizing healthcare during the last 3 months before the interview. We found a higher frequency of visits to informal than formal healthcare providers in both states, but also a higher frequency in Anambra than in Enugu (71% vs. 47%). The most frequent health conditions reported among household members were communicable diseases, accounting for 90% (N = 925) of the cases. The data indicates that most health-seeking behavior involved outpatient services, with 93% (N = 436) in Anambra and 96% (N = 399) in Enugu.

Challenges to access healthcare included the high cost of treatment (Anambra: 78%; Enugu: 75%), lack of money to pay for treatment (Anambra: 65%; Enugu: 68%), and lack of drugs in government facilities (Anambra: 52%; Enugu: 53%) (Table 3).

**TABLE 3:** Characteristics of healthcare access and utilization (Enugu and Anambra, Nigeria. 2022).

Variables	Anambra N = 509	Enugu N = 516	Overall N = 1,025
Facility type sought^a^ ^b^, N (%)			
Formal Public	106 (24.20)	71 (13.76)	177 (17.27)
Formal Private	198 (38.90)	117 (22.67)	315 (30.73)
Informal Provider	363 (71.32)	242 (46.90)	605 (59.02)
Not specified^c^	29 (5.70)	16 (3.10)	45 (4.39)
Not utilized	39 (7.66)	94 (18.22)	133 (12.98)
Health conditions^a^ ^d^, N (%)			
Noncommunicable diseases	16 (3.14)	11 (2.13)	27 (2.63)
Communicable diseases	473 (92.93)	452 (87.60)	925 (90.24)
Child and maternal healthcare	146 (28.68)	85 (16.47)	231 (22.54)
Unspecified health conditions^a^ ^e^	188 (40.69)	55 (13.78)	243 (28.22)
Nature of care, N (%)^f^			
Outpatient visits only	436 (93.36)	399 (95.91)	835 (94.56)
In-patient visit only	17 (3.64)	12 (2.88)	29 (3.28)
Outpatient and inpatient	14 (3.00)	5 (1.20)	19 (2.15)
Main challenges with access to health facilities^a^			
High cost of treatment	399 (78.39)	386 (74.81)	785 (76.59)
Non-availability of government health facilities	161 (31.63)	190 (36.82)	351 (34.24)
Lack of money to pay for treatment	333 (65.42)	352 (68.22)	685 (66.83)
Lack of drugs in government facilities	264 (51.87)	275 (53.29)	539 (52.59)
Difficulty with transportation to health facilities	91 (17.88)	74 (14.34)	165 (16.10)

^a^ Multiple choice.

^b^ Formal public: *PHC, hospital;* Formal private: private hospital, pharmacy shops, and medical laboratory; Informal provider: PMV, and herbalists.

^c^ Not specified: provider type is not clearly described. Not utilized: were not sick.

^d^ Noncommunicable diseases: diabetes, cancer, and hypertension; communicable diseases: malaria, respiratory tract infection, and diarrhea; Child/maternal health: immunization services, antenatal care, and childbirth.

^**e**^ Unspecified health conditions: pain/ache in general, skin rash, not specified eye problems.

^f^ Missing data = 9.

### The Association Between OOPE in Health, Socio-Economic Status, and Health-Seeking Behavior of Households Living in Urban Slums in Both States

Table 4 shows the association between OOPE and socio-economic status, and healthcare utilization characteristics. Residents of urban slums in Enugu spent less on OOPE compared to those in Anambra (mean difference: −2,854; 95% CI: −4,241; −1,466). Individuals with formal occupations (e.g., government workers, employees in the private sector, etc.) also had lower OOPE compared to those in informal occupations (e.g., artisans, subsistence farmers, petty traders) (mean difference: −1,296; 95% CI: −2,570; −21). Households in the middle wealth quintile incurred higher costs than those in the poorest wealth quintile (mean difference: 2,389; 95% CI: 160; 4,619). No other variables showed statistically significant differences.

**TABLE 4:** Association between out-of-pocket expenditure (in Nigerian Naira) and socio-economic characteristics (Enugu and Anambra, Nigeria. 2022).

Variables	Mean OOPE, NGN (95% CI)	Mean difference (95% CI)
State		
Anambra	5,437 (4,163; 6,711)	Ref
Enugu	2,583 (2,033; 3,133)	−2,854 (−4,241; −1,466)
Age		
<26	3,616 (1,856; 5,376)	Ref
26–35	3,603 (2,618; 4,588)	−12 (−1,560; 1,534)
36–45	5,169 (2,144; 8,193)	1,552 (−2,251; 5,356)
45 above	3,878 (2,130; 5,626)	262 (−1,653; 2,177)
Occupation		
Informal	4,187 (2,760; 5,613)	Ref
Unemployed	3,415 (1,235; 5,595)	−771 (−3,158; 1,615)
Formal	2,891 (2,103; 3,678)	−1,296 (−2,570; −21)
Highest Education		
Primary education and less	4,545 (2,397; 6,693)	Ref
Secondary education	3,681 (2,884; 4,478)	−863 (−2,763; 1,036)
Tertiary education	5,574 (4,281; 0720)	1,029 (−4,295; 6,354)
Marital Status		
Single	3,454 (1,439; 5,470)	Ref
Married	4,040 (2,763; 5,318)	585 (−1,326; 2,498)
SES Quintiles		
Poorest	3,302 (2,180; 4,426)	Ref
Less Poor	4,163 (2,802; 5,525)	860 (−413; 2,133)
Middle	5,692 (2,823; 8,561)	2,389 (160; 4,619)
Rich	3,991 (2,398; 5,584)	688 (−685; 2,061)
Richest	2,854 (1,890; 3,817)	−448 (−1,572; 674)
Facility Type Sought		
Formal healthcare facility only	5,194 (914; 9,474)	Ref
Informal healthcare facility only	3,281 (2,519; 4,042)	−1,913 (−6,211; 2,384)
Both formal and informal healthcare facility	5,315 (4,138; 6,492)	121 (−4,049; 4,290)

Exchange rate USD/Nigerian Naira, September 2022: 1 USD: 430.99 NGN.

### Inequity in Healthcare Expenditure Among Households Living in Urban Slums

**FIGURE 1:**
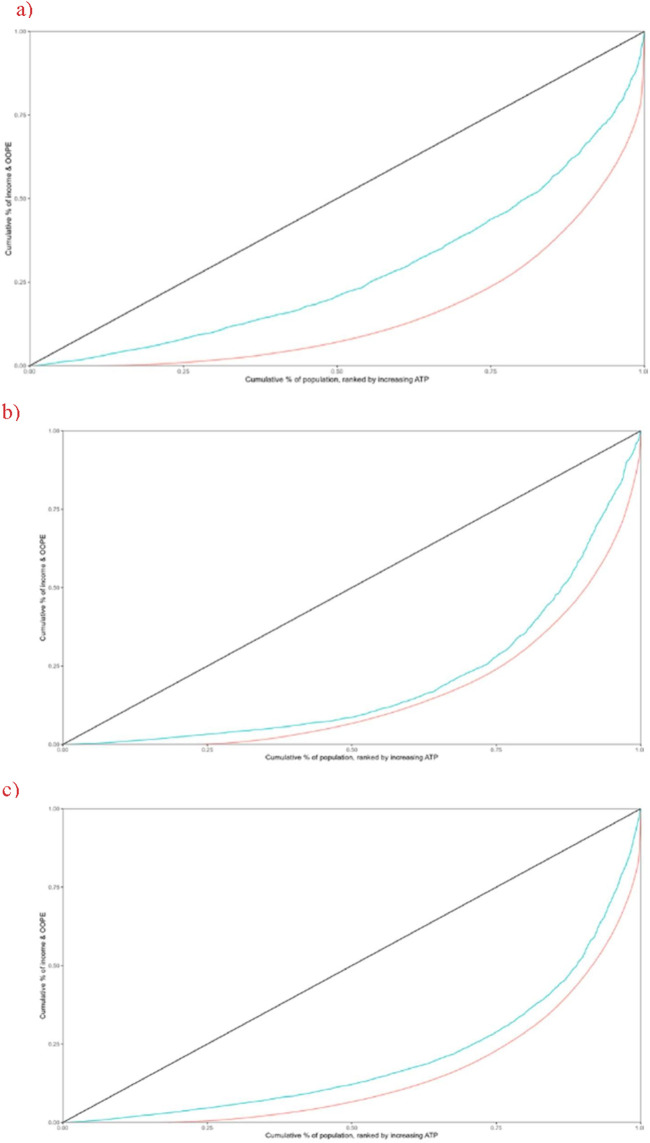
Concentration and Lorenz curves of ability to pay (Enugu and Anambra, Nigeria. 2022). The red line indicates the Concentration curve, the blue line indicates the Lorenz curve of ATP and the black line indicates the line of equality. **(A)** Anambra. **(B)** Enugu. **(C)** Pooled sample.

The financing incidence analysis indicated a progressive pattern of OOPEs in Enugu and Anambra. The Gini index represented by the Lorenz curve of ATP indicated some concentration of ability to pay (ATP) among the rich across the wealth quintiles. (Anambra: 0.428, Enugu: 0.572, All: 0.585). The concentration index obtained from the concentration curve shows a progressive pattern of OOPE where healthcare payments are concentrated amongst the richest households (Anambra: 0.687, Enugu: 0.678, All: 0.695). The Kakwani index also indicated a more progressive financing system in terms of OOPE, with the richest contributing proportionately more than their share of ATP (Anambra: 0.259; Enugu: 0.103; All: 0.110) (Figures 1A–C).

**FIGURE 2:**
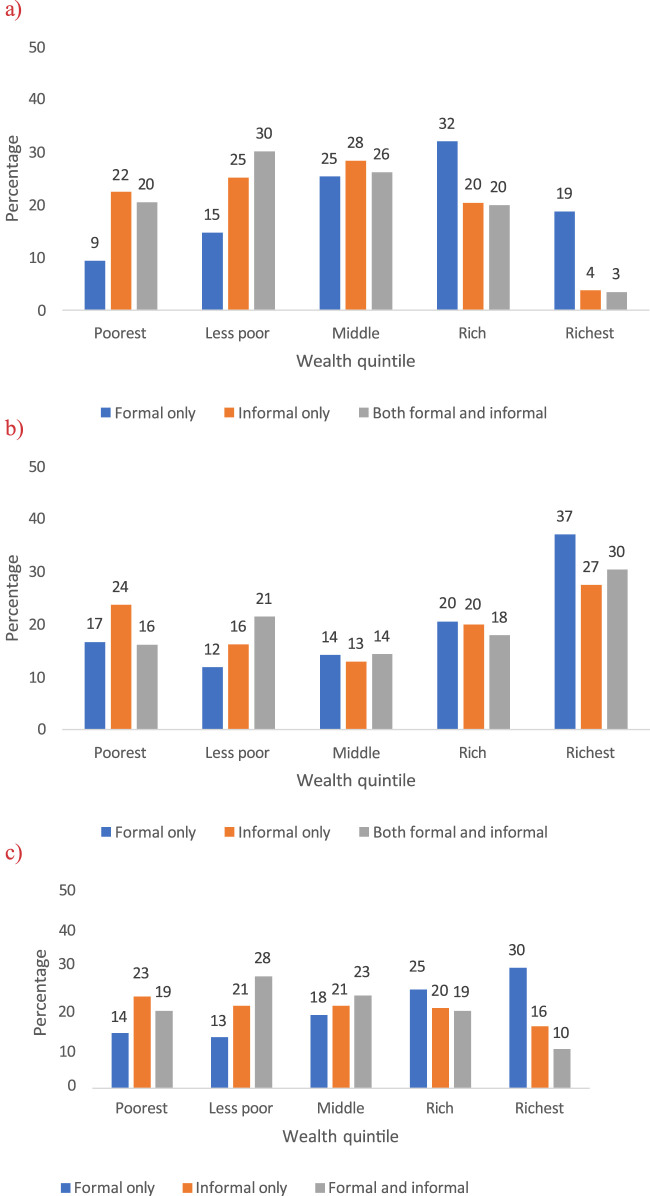
Type of health provider accessed according to the wealth quintile (Enugu and Anambra, Nigeria. 2022). **(A)** Anambra. **(B)** Enugu. **(C)** Pooled sample.

When examining the relationship between the wealth quintile and the type of health services sought, the findings indicated that wealthier households used formal healthcare services more often than the poorest households. In Anambra, the richest households used formal services 2.1 times more than the poorest households, and in Enugu, this usage was 2.2 times higher. Conversely, the poorest households used informal health services more frequently than the richest households in Anambra (6.7 times higher) and overall (1.9 times higher). In Enugu, there was an increasing trend in the utilization of all types of health providers by the richest quintiles compared to the poorest, with usage 2.2, 1.1, and 1.9 times higher for formal, informal, and combined formal/informal health services, respectively (Figure 2).

## Discussion

This study examined the economic burden and inequity in OOPEs on healthcare in urban slums in Enugu and Anambra States, Nigeria. The findings showed a high economic burden among specific socio-economic groups and study sites. The financing incidence analysis indicated a progressive pattern, with the wealthiest quintiles contributing the most OOPE to the health system. Further inquiry reveals that the progressivity in OOPE can be explained by the higher use of informal healthcare facilities by the poorest households.

### OOPE

Our study revealed important differences in the economic burden of healthcare access across study sites and socio-economic groups. We found that residents of Anambra employed in the informal market and the middle wealth quintile are more at risk of incurring high OOPE when accessing healthcare. These findings are consistent with other studies, showing that OOPE varies across slum sites and socio-economic groups [14, 32–35]. For example, previous studies in Bangladesh and Sierra Leone demonstrated differences in OOPE among slums [14, 34]. This is due to some slums being more centrally located and thus easier to access healthcare, or some slums having better healthcare provision than others, which reduces transportation costs. In this study, the higher OOPE among residents employed in the informal market suggests a lack of safety net and poorer health compared to those with formal jobs [35]. However, evidence on the vulnerability to high OOPE by job category is still limited and needs further investigation to guide policies protecting informal workers from further impoverishment due to healthcare utilization.

Recent studies examining the economic burden of healthcare access and utilization in LMICs have highlighted the significant financial challenges faced by slum dwellers when seeking medical care. These studies indicate various socio-economic and healthcare characteristics associated with high OOPE and catastrophic costs. In Nigeria, the incidence of catastrophic expenditure among individuals scheduled for emergency surgeries was notably high among slum dwellers, reaching 74.1% [36]. Multiple logistic regression models also identified that slum dwellers were more likely to incur catastrophic expenditure than non-slum dwellers [36]. In Sierra Leone, a cross-sectional survey conducted in three slum settlements in urban Freetown revealed that slums situated in central areas experienced lower direct medical, non-medical, and total costs when accessing services outside their communities compared to those located in hilly regions and/or farther from the city center [37].

Taken together, slum settlements are diverse, leading to varying vulnerabilities among residents when accessing healthcare. Policymakers should consider these diversities in the attempt to improve healthcare access for those who are most at risk of falling deeper into poverty due to the high costs associated with healthcare.

### Inequities in Healthcare Access

Regarding the distribution of OOPE across wealth quintiles, the concentration, and Kakwani indices indicated expenditures concentrated among the richest quintiles or a progressive pattern. A study using data from the Sierra Leone Integrated Household Survey (2018) provides insights into the health financing system in Sierra Leone, specifically regarding its progressivity and regressivity. The findings indicate that primary healthcare is pro-poor (progressive); however, when OOPE for health is considered, the overall health financing system becomes regressive due to the regressive nature of these expenditures [38]. It is important to note that this study does not account for slum dwellers, focusing solely on the national scenario.

The degree of regressivity and progressivity in healthcare spending plays a crucial role in achieving universal health coverage, especially in countries like Nigeria, where OOPE is the primary source of health financing. Relying heavily on OOPE can create significant disparities in healthcare spending, further deteriorating health outcomes for the most vulnerable individuals living in urban slums.

More reflections are also necessary when discussing progressivity and regressivity in OOPE. Although the progressivity in OOPE indicates a fair health system as the richest contribute the most, it can also indicate a lack of access to good quality and usually more expensive formal health services [3, 32]. Indeed, our findings indicated that poorer households in the slums often seek cheaper services from the informal sector rather than opting for the more expensive but higher-quality care offered by the formal sector. This explains the observed progressivity in the current study. Essentially, poorer households resort to the services of informal providers and are relatively priced out of more optimal healthcare services from the formal providers by the wealthier households who can relatively afford them. Meanwhile, the poorer households resort more to seeking cheaper and less standard services from informal providers since they are more affordable [2, 39]. These findings amplify the importance of the observation made by Ataguba et al. [3] that progressivity does not always indicate fairness and equity.

In addition, since our study was conducted in slums and the wealth quintiles were defined based on the survey sample rather than national estimates, it is crucial to emphasise that wealthier households in our study would still be classified as urban poor. Informal urban areas have historically been deprived of basic services and face inadequate housing conditions, making slum dwellers more vulnerable than those in formal urban settings [40]. Consequently, while the Gini index suggests some concentration of ATP among the rich, these better-off households may still suffer inequities in healthcare access and subsequent health outcomes. Such inequity has been argued to hinder individual and community growth in slums [41].

### Implications of This Study and Policies to Mitigate the Economic Burden

Our findings are important as they provide key information for policymakers to understand and identify specific socio-economic groups among the slum dwellers that interventions geared towards alleviating the burden of OOPE on health should target most. This will also increase the chances that the intervention is effective since those who receive it are those who need it most.

Given the scarcity of research evaluating the inequities in OOPE for health among urban slum dwellers, our findings help to establish a crucial empirical foundation. These results address a significant gap in the literature and offer an essential starting point for researchers interested in the complexities of inequity in health-related OOPE expenses within slum communities.

Our study serves as an invitation for more investigation, encouraging researchers to deepen their understanding of the nuanced factors contributing to and shaping the landscape of OOPE in slums by further exploring the extent to which the ability to pay for healthcare can price out the urban poor, hence denying them access to essential care services. This enhances the comprehensiveness of research in this domain and fosters a more informed and targeted approach toward developing interventions and policies to promote equity in healthcare access and financial burdens among slum dwellers.

Future research is needed to determine how policies such as health insurance, linkages between formal and informal providers, and improvement of the Basic Healthcare Provision Fund (BHCPF) could help reduce the burden of OOPE among households in urban slums in Nigeria.

In terms of health insurance, NHIS coverage is very limited in Nigeria (∼3% of the population). Studies conducted by Gustafsson-Wright et al. [42] and Okunogbe et al. [43] indicated that health insurance schemes can be an effective tool in mitigating the impact of OOPE on health, highlighting the importance of adequate healthcare insurance coverage. Ensuring that more urban slum dwellers, especially those without formal occupation as well as the poorest households, are enrolled in health insurance schemes could be an effective way to reduce the impact of OOPE.

Given that poorer households are more likely to use informal services, creating linkages between formal and informal providers could also be effective in improving the quality of health services and reducing OOPE in Nigeria and other LMICs. Evidence from Nigeria indicates that stakeholders have a positive view about linkages between formal and informal health services. The hypothesis is that it could lead to quality improvement in service delivery. The study suggested that collaboration with the informal sector can be implemented through regulatory and fiscal measures, improvement of clinical guidelines, and engagement with the communities [44]. A study by Ozor et al. [45] in 2024, showed that collaboration between formal and informal health providers is a key factor in strengthening and ensuring quality healthcare service provision in Nigerian communities. Another study in Sierra Leone showed that regulating the informal sector could contribute to managing non-communicable diseases. Community health workers suggested collaboration with traditional healers to identify and refer hypertensive patients [46].

Despite the positive findings, evidence also highlights the challenges and risks associated with integrating informal providers into the formal health system in LMICs. For instance, a study in India that examined the incorporation of informal providers into the National Tuberculosis (TB) Program raised concerns about the low quality of services offered [47]. Similarly, in China, seeking treatment from informal providers often results in missed or delayed diagnoses, which can lead to severe clinical consequences [48]. Therefore, we recommend a comprehensive implementation strategy that includes sufficient resources, active community participation, and regular evaluation and monitoring mechanisms when linking informal health providers with the formal health system.

Our study demonstrates that inequity in access to healthcare remains prominent in urban slums in Nigeria. Consequently, improving the Basic Healthcare Provision Fund (BHCPF) will be pivotal to reducing the economic impact and inequities in healthcare access for the urban poor in Nigeria. The fund was established under section 11 of the Nigerian National Health Act in 2014 as a catalyst to improve access to primary healthcare, especially for the urban poor. It funds a Basic Minimum Package of Health Services, increases the fiscal space for health, and strengthens the national health system. The mechanism is based on providing routine daily operation costs of primary healthcare and ensuring access to healthcare for all [49]. Modeling studies could indicate the impact of BHCPF on reducing the economic impact of healthcare access among urban slum dwellers.

### Strengths and Limitations

This study was led by the Community-Led Responsive and Effective Urban Health Systems (CHORUS) research, which engaged closely with communities, health professionals, and city-level decision-makers to develop and test ways to improve the health of the poorest urban residents. The survey was the first study focusing on the economic burden and healthcare access inequities in urban informal settlements in Enugu and Anambra States. It provides reference material for decision-making policies to address challenges slum dwellers face when seeking healthcare.

Our study also has some limitations. The financing incidence analysis included only OOPEs, excluding tax revenue, donor funding, and health insurance (community and social). However, as most of the health system is financed out-of-pocket (69%), our analysis provides a good picture of the inequities in healthcare access. Our analysis relied on self-reported health data, which can be subject to reporting bias, particularly regarding costing data. To minimize bias, a short time horizon to report costs was applied. Finally, a short timeframe may also risk minimizing the costs associated with lengthy pathways to care for chronic health conditions.

### Conclusion

Our study explored the economic burden and inequities of OOPE among urban slum dwellers in Nigeria. We found that healthcare costs vary widely, particularly for residents of Anambra State and those employed in the informal sector, who face higher expenses. Although OOPE for healthcare in urban slums in Enugu and Anambra states is progressive, this is mainly driven by the lack of access to quality and formal healthcare by the poorest rather than fair payment for health services. Our findings underscore the disparities in access to quality healthcare in urban slums in both states and call for more attention to the plight of the urban poor in the states.

The findings also highlight the role that ATP may play in the lack of access to quality healthcare among the poorest who are priced out of the system as well as the households in socio-economic groups that are most affected by OOPE in health. Therefore, there is a need for further investigations into the causes of inequities in urban slums. We need to assess the feasibility, costs, and cost-effectiveness of integrating informal health providers into the formal health system. Additionally, conducting a needs assessment will help gather the necessary information to implement changes that will be beneficial for the health of the urban poor.
