# Supplementary material for: Inequities in Household Out-Of-Pocket Spending Among Urban Slum Dwellers in Southeast Nigeria
Source: Int J Public Health. 2025 Mar 27;70:1607969. doi: 10.3389/ijph.2025.1607969 (PMC11981907; doi:10.3389/ijph.2025.1607969)
Supplement: Supplementary file 1 [file Image1.pdf]

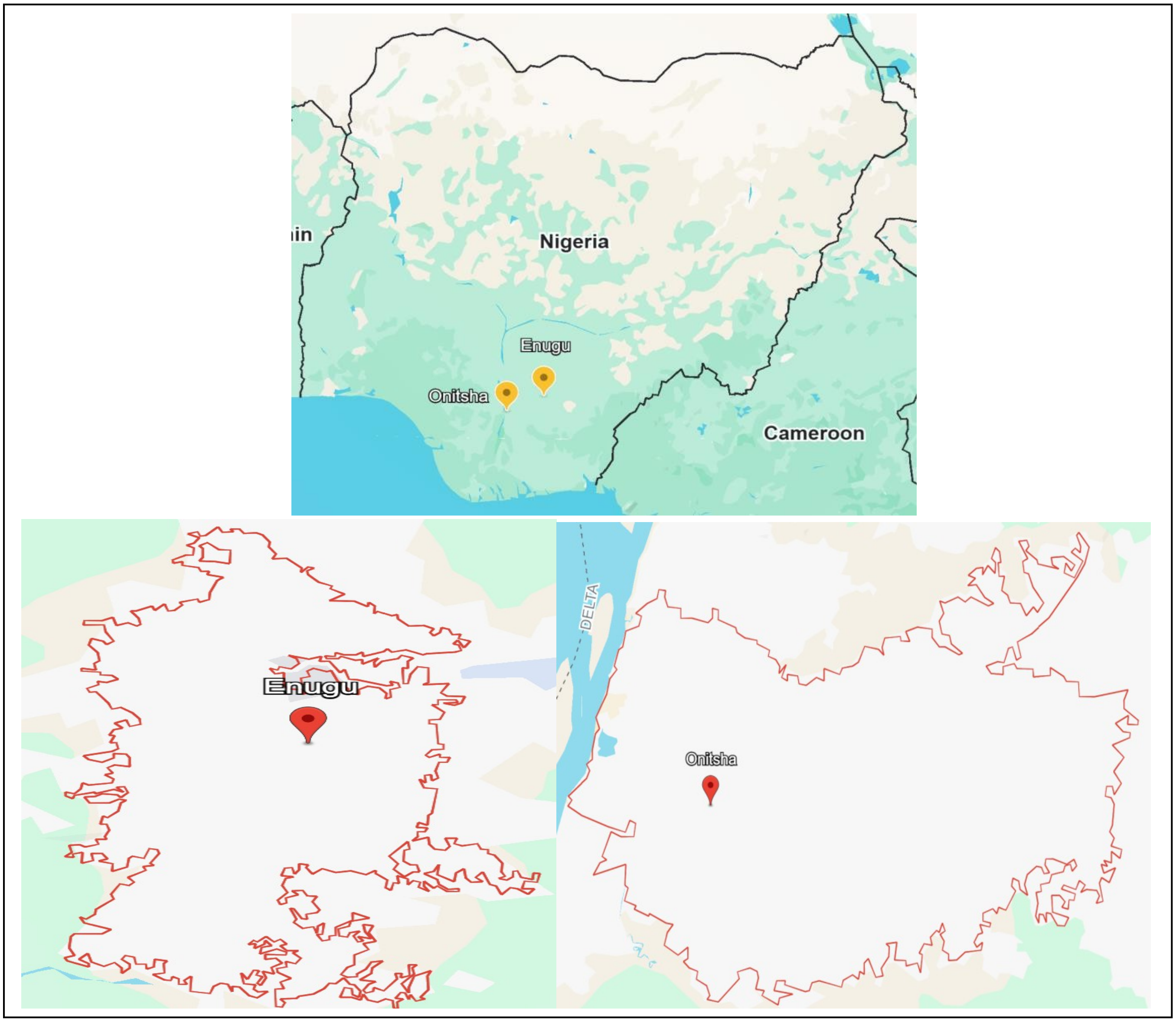

Supplementary File 1: Map of the study sites: Google Earth Version 7.3.6.10155. (2024.12.20). Nigeria (9°48'56" N, 8°04'12" E), Enugu (6°40'47" N, 7°29'49" E), Onitsha (6°21'40" N, 6°48'12" E), Image by Sushan Li, https://earth.google.com/web/, (2024.12.20) **(Enugu and Onitsha, Nigeria. 2024)**
